# Association between HbA1c Levels and Fetal Macrosomia and Large for Gestational Age Babies in Women with Gestational Diabetes Mellitus: A Systematic Review and Meta-Analysis of 17,711 Women

**DOI:** 10.3390/jcm12113852

**Published:** 2023-06-05

**Authors:** Sudipta Sarker Mou, Clare Gillies, Jiamiao Hu, Marianna Danielli, Bassel Hamameeh Al Wattar, Kamlesh Khunti, Bee Kang Tan

**Affiliations:** 1 Department of Cardiovascular Sciences, University of Leicester, Leicester LE1 7RH, UK; 2 Diabetes Research Centre, Leicester General Hospital, Leicester LE5 4PW, UK; 3 Engineering Research Centre of Fujian-Taiwan Special Marine Food Processing and Nutrition, Ministry of Education, Fuzhou 350002, China; 4 St Helier Hospital, Epsom and St Helier University Hospitals NHS Trust, Carshalton SM5 1AA, UK

**Keywords:** gestational diabetes mellitus, GDM, glycated haemoglobin, HbA1c, fetal macrosomia, large for gestational age, LGA

## Abstract

Gestational diabetes mellitus (GDM) is the most common metabolic disorder in pregnancy. GDM is associated with serious maternal and fetal complications, in particular, fetal macrosomia and large for gestational age (LGA), which predisposes to a higher risk of childhood obesity and type 2 diabetes mellitus later in life. Early prediction and diagnosis of GDM leads to early interventions such as diet and lifestyle, which could mitigate the maternal and fetal complications associated with GDM. Glycated haemoglobin A1c (HbA1c) has been widely used for monitoring, screening for and diagnosing diabetes and prediabetes. Increasing evidence has also showed that HbA1c could indicate fetal glucose supply. Thus, we hypothesise that the HbA1c level at around 24 to 28 weeks may predict the development of fetal macrosomia or an LGA baby in women with GDM, which could be useful for better prevention of fetal macrosomia and LGA. We searched MEDLINE, EMBASE, Cochrane and Google Scholar databases from inception to November 2022 for relevant studies that reported at least one HbA1c level during 24–28 weeks of pregnancy and fetal macrosomia or an LGA baby. We excluded studies that were not published in the English language. No other search filters were applied during the search. Two independent reviewers selected eligible studies for meta-analysis. Two independent reviewers performed data collection and analyses. The PROSPERO registration number is CRD42018086175. A total of 23 studies were included in this systematic review. Of these, 8 papers reported data of 17,711 women with GDM that allowed for inclusion in a meta-analysis. The obtained results demonstrated the prevalence of fetal macrosomia was 7.4% and of LGA, 13.36%. Meta-analyses showed that the estimated pooled risk ratio (RR) for LGA in women with high HbA1c values compared to normal or low values was 1.70 (95% CI: 1.23–2.35), *p* = 0.001; and the pooled RR for fetal macrosomia was 1.45 (95% CI: 0.80 to 2.63), *p* = 0.215. Further research is needed to evaluate the utility of HbA1c levels in predicting the delivery of a baby with fetal macrosomia or LGA in pregnant women.

## 1. Introduction

Gestational diabetes mellitus (GDM) is the most common metabolic complication in pregnancy, affecting around 7–10% of pregnant women worldwide [1,2]. GDM is defined as hyperglycaemia that first appears during pregnancy, especially in the second half of gestation. GDM increases the risk of several pregnancy complications affecting mothers and their babies. Fetal macrosomia is one of the most typical fetal abnormalities associated with GDM [3], affecting around 15–45% of babies born to women with GDM [4]. Women with GDM are at an increased risk of caesarean delivery [4], pre-eclampsia, preterm delivery and stillbirth. Fetal hypoglycaemia, shoulder dystocia, metabolic disturbances after birth [5], obesity and diabetes during childhood and adulthood are also prevalent. Early diagnosis of fetal macrosomia could help optimise the delivery time and reduce the risk of adverse outcomes [6,7,8]. Furthermore, women who develop GDM have a ten-times increased risk of developing type 2 diabetes mellitus later in life. Once GDM is diagnosed in one pregnancy, there is an increased risk of the development of GDM in subsequent pregnancies. The offspring from women with GDM also have a higher risk of developing type 2 diabetes mellitus during adulthood. Therefore, this vicious cycle continues and increases the burden of this global pandemic.

GDM diagnosis differs between countries. Some countries use their own guidelines, while others use internationally recognised guidelines, such as the IADPSG, ADIPS, ADA, NICE, Carpenter and Coustan criteria, and WHO guidelines. The ADIPS guideline has been popular since 1998. However, new guidelines from IADPSG were published in 2015 with new diagnostic criteria for GDM. According to IADPSG, women are diagnosed with GDM if they have a fasting plasma glucose of 5.1 mmol/L or a 2 h 75 g Oral Glucose Tolerance Test (OGTT) plasma glucose of 8.5 mmol/L. The IADPSG diagnostic criteria has substantially increased the prevalence of GDM [9].

The OGTT is the established test used to diagnose GDM. A standard dose of 75 g of glucose oral solution is ingested and blood glucose levels are checked after two hours [10]. The OGTT determines how quickly glucose is cleared from the maternal circulation. However, there are several known limitations of the OGTT [11]. Firstly, the OGTT is inaccurate in non-obese or critically ill patients [12]. Another critical limitation is that the OGTT test process is lengthy and inconvenient for the patient. Patients need to fast for a minimum of eight hours before the test, and several blood samples are collected from the patient during the test, which could be challenging for some patients.

Glycated haemoglobin A1c (HbA1c) is an established screening and diagnostic test for diabetes mellitus. HbA1c reflects the average blood glucose level over the past two to three months. An HbA1c level below 5.7% is considered normal. The range between 5.7% and 6.5% is considered prediabetes, and a level above 6.5% is indicative of overt diabetes. During pregnancy, physiological hemodilution, increases in erythropoiesis and alterations in red blood cell kinetics may further influence HbA1c levels. Therefore, the usefulness of HbA1c in diagnosing GDM remains debatable [13]. According to the NICE guidelines, pregnant women with diabetes should aim to control their HbA1c to below 6.1% (43 mmol/mol). Notably, it is generally accepted that HbA1c is a key parameter reflecting the fetal glucose supply, which is highly likely to have an impact on infant size at birth. Therefore, we hypothesise that HbA1c at around 24 to 28 weeks may predict the development of fetal macrosomia or a large for gestational age (LGA) baby in women with GDM.

It is essential to identify those who are at a higher risk of developing GDM and its associated complications, in particular fetal macrosomia or an LGA baby. Once identified, several treatment options can be provided to achieve reasonable glycaemic control, which could eventually reduce the incidence of fetal macrosomia or LGA babies. This systematic review aimed to determine the association between high HbA1c levels and fetal macrosomia or LGA in women with GDM. To the best of our knowledge, this the first systematic review and meta-analysis that studied the association between HbA1c and fetal macrosomia or LGA in women with GDM.

## 2. Material and Methods

We conducted this systematic review according to the Preferred Reporting Items for Systematic Review and Meta-Analysis (PRISMA) and the Meta-Analysis of Observational Studies in Epidemiology (MOOSE) guidelines [14]. The protocol was registered with the International Prospective Register for Systematic Reviews (PROSPERO) database; the registration number is CRD42018086175.

### 2.1. Search Strategy and Selection Criteria

We searched MEDLINE, Embase, Cochrane and Google Scholar databases from inception to November 2022. The search strategy for each database was developed using the following MeSH terms and keywords: “gestational diabetes”, “pregnancy”, “fetal macrosomia”, “large for gestational age (LGA) and “glycated haemoglobin (HbA1c)”. The search terms were combined using Boolean operators. There was no limit on the publishing year, but only human studies in the English language were included. We also included all observational studies that assessed pregnant women with GDM, with at least one HbA1c measurement performed around 24–28 weeks of gestation and which reported fetal macrosomia or LGA as an outcome. Fetal macrosomia is defined as a birth weight of more than 4.0 kg [4]. LGA is defined as a fetal size greater than the 90th percentile. Exclusion criteria consisted of women with type 1 or type 2 diabetes mellitus; studies where GDM was diagnosed by OGTT only, with no HbA1c undertaken; conferences or meetings where no full article was found; and studies where outcomes were reported or compared on the effect of different treatment options for women with GDM and where the results of interest were not reported by HbA1c levels. Studies that reported no primary data, case reports, and case studies were also excluded. The search strategy was formulated by the first author (S.S.M.) with the help of a librarian, and then reviewed by C.L.G. and B.K.T. A copy of the search strategy has been added to the Supplementary Material.

In this systematic review, the following studies were included:

- Any study design addressing the research question,
- Including women with GDM diagnoses at any point in pregnancy (singleton pregnancy),
- Including women who received HbA1c during their pregnancy and reported the time of testing,
- Reported on fetal macrosomia or birth weight,
- If birth weight is reported as a dichotomous outcome (primary or secondary), a threshold of 4 kg was used to define fetal macrosomia.

The following studies were excluded:

- Women with type 1 or type 2 diabetes,
- GDM being diagnosed on OGTT only with no HbA1c,
- Large for gestational age reported with no clear definition of fetal macrosomia or birth weight thresholds,
- Studies with no primary data and/or case report and case series,
- Studies in animals.

### 2.2. Outcome Measures

The primary outcome measure was the number of fetal macrosomia or LGA babies delivered by women with GDM. Secondary outcome measures were maternal pre-pregnancy BMI and maternal age.

### 2.3. Data Collection

The first author (S.S.M.) combined all the search results in Endnote and removed duplicates. Two individual reviewers (S.S.M. and M.D.) carried out a screen of the titles and abstracts separately, according to the eligibility criteria mentioned in the inclusion and exclusion criteria. The studies that did not meet the inclusion criteria or were irrelevant to the research question were removed. Full texts of the remaining studies were obtained for further scrutiny to determine whether they met the inclusion criteria. Reference lists from eligible articles were also checked for additional studies. Any disagreements were resolved through discussion with a third reviewer (B.K.T.).

We used a standardised data extraction form to extract data from the included articles. Data were collected under the following headings: first author, year of publication, country, the total number of women with GDM, number of fetal macrosomia, number of LGA babies, mean HbA1c, GDM diagnostic criteria, duration of the study, mean maternal age and mean body mass index (BMI). We created a separate spreadsheet to extract the number of LGA babies or macrosomic babies in women with high HbA1c levels (as defined by the study), and in women with normal or low levels. Data extraction was performed independently by two researchers (S.S.M. and J.H.) and any discrepancies were resolved after discussing with C.L.G. and B.K.T.

### 2.4. Data Analysis

We employed a random-effects meta-analysis model to pool study results from the included articles. The model reported the prevalence, risk ratios (RRs) and their 95% CIs from the included studies. Heterogeneity between studies was assessed using the I-squared statistic [15]. Funnel plots and Egger’s tests were conducted to evaluate publication bias. A *p*-value of 0.05 or less was considered significant. All statistical analyses were performed using Stata 16 (StataCorp., College Station, TX, USA).

### 2.5. Assessment of the Risk of Bias

We used the National Institute of Health (NIH) assessment tool for the observational cohort to check the quality of selected papers [16]. The NIH assessment tool has a total score of fourteen. Studies which scored ten or above were considered good quality studies. We used the I-squared and Chi-squared tests to assess study heterogeneity.

## 3. Results

### 3.1. Search Results and Characteristics of Included Studies

**Figure 1:**
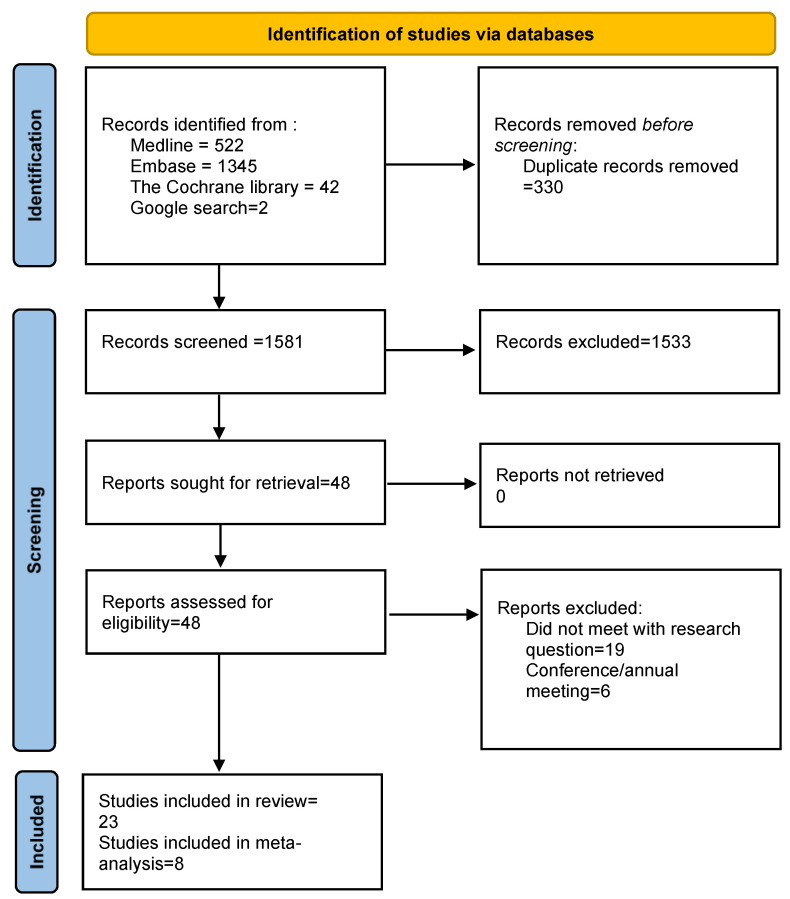
Preferred Reporting Items for Systematic Reviews and Meta-Analyses (PRISMA) chart of study selection for systematic review and meta-analysis.

Figure 1 contains the Preferred Reporting Items for Systematic Reviews and Meta-Analyses (PRISMA) flow diagram for our search strategy. Our systematic search identified a total of 1911 papers. Of those, 330 duplicates were removed, and 1581 titles and abstracts were reviewed. Articles that did not match the research question, meeting abstracts, papers that did not have at least one HbA1c measurement or did not report fetal macrosomia or LGA were excluded. We selected 48 articles for full-text review. We excluded a further 25 articles, as they did not have the data required for our systematic review. The remaining 23 articles were selected for inclusion in our systematic review. Reference lists from these 23 papers were investigated for additional articles. However, no other relevant articles were found. Finally, after carefully reviewing the data presented in these 23 articles, we had 8 articles for the meta-analysis. Amongst the eight articles in the meta-analysis [17,18,19,20,21,22,23], six studies reported the percentages of LGA babies stratified by high or low HbA1c levels, and four studies reported numbers of fetal macrosomia and HbA1c levels. Two studies, i.e., Sweeting et al. [19] and Katon et al. [21], reported both outcomes. The cut-off value for HbA1c for these eight studies, ranged from 5 to 6.5.

Table 1 summarises the characteristics of the included studies. All the included studies were observational. Eight studies were described as retrospective, three as prospective and the remaining studies were described as observational studies. Studies were conducted in different countries, i.e., four studies were from China [24,25,26,27], three studies from Italy [28,29,30], two studies each from Saudi Arabia [31,32], Australia [19,20], USA [21,33], and Spain [17,34], and one study each from Brazil [35], Turkey [36], Romania [37], Switzerland [38], Singapore [22], Chile [18], Denmark [23] and North Macedonia [39]. All of the studies measured HbA1c around 24–28 weeks of pregnancy and took place in a hospital setting. Various diagnostic criteria were used in the diagnosis of GDM. Three studies used the IADPSG criteria, four used the Carpenter and Coustan criteria, two used the ADA criteria, two used the ADP criteria, four used the WHO criteria, one each used the ADIPS, NDDG, 2 h OGTT, 75 g OGTT, respectively. Eighteen studies reported the fetal outcomes as fetal macrosomia, and fifteen studies reported as LGA babies. Publication year ranged from 2007 to 2020. On further scrutinising these articles, the oldest data was from 1987 [17], and the newest data was from 2018 [36]. Study populations were of mixed ethnicity. The studies had mean maternal ages ranging from 30.9 years to 36.6 years, and mean BMI ranging from 22.02 to 32.3. The smallest study had 22 women with GDM and the largest study had 3218 women with GDM. Six studies had less than 100 participants and eight had more than 1000 study participants. Finally, Table 2 describes the funding source of the included studies.

In this systematic review, we assessed a total of 17,711 women with GDM; 1317 had fetal macrosomia and 2367 had an LGA baby. In most of the included studies, the mean HbA1c was above 5.5%. Further, women with GDM who had an HbA1c of more than 5.5% at around 24–28 weeks of gestation had higher chances of delivering a baby with fetal macrosomia or an LGA baby. In addition, the mean maternal age was above 30.9 years and the mean pre-pregnancy BMI was above 24 kg/m^2^ with the exception of two studies, Liu et al. and Hu et al. [24,25], suggesting that maternal age and pre-pregnancy BMI and maternal age in women with GDM may contribute to the development of fetal macrosomia or an LGA baby.

### 3.2. Meta-Analysis

**Figure 2:**
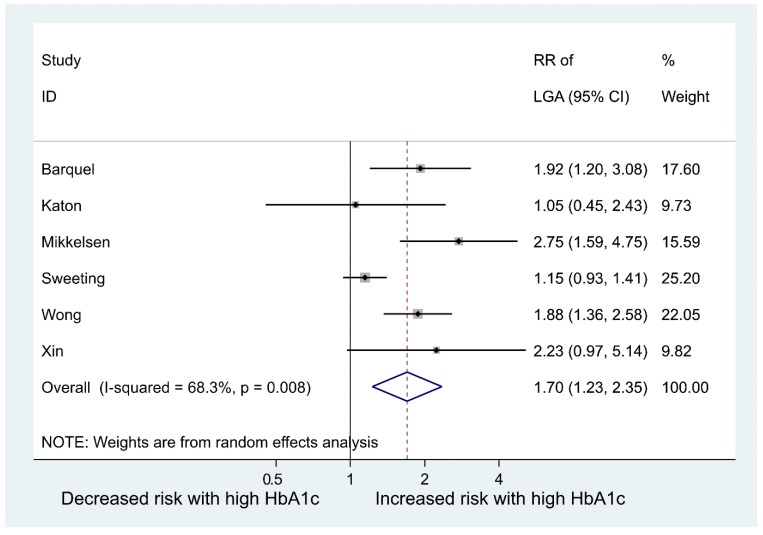
Risk ratio of having a large for gestational age (LGA) baby in women with gestational diabetes mellitus (GDM) who have high vs. normal or low HbA1c levels.

**Figure 3:**
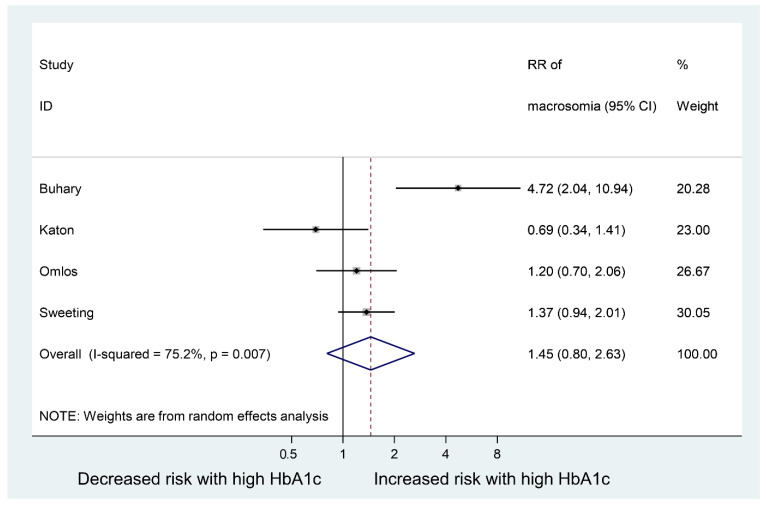
Risk ratio of having a baby with fetal macrosomia in women with gestational diabetes mellitus (GDM) who have high vs. normal or low HbA1c levels.

**Figure 4:**
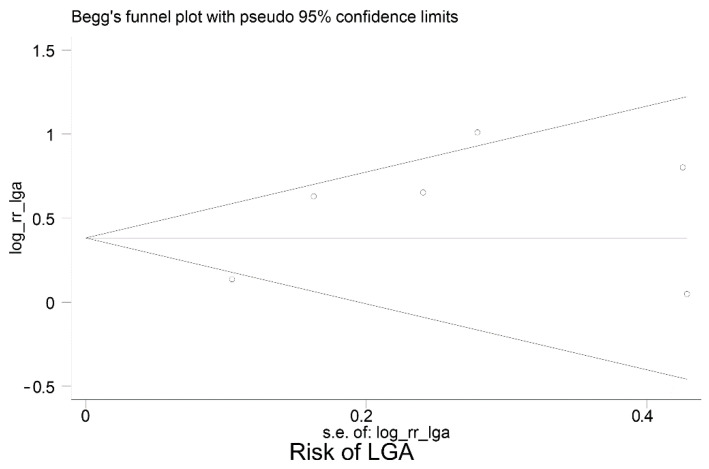
Funnel plot for publication bias—Large for Gestational Age (LGA).

**Figure 5:**
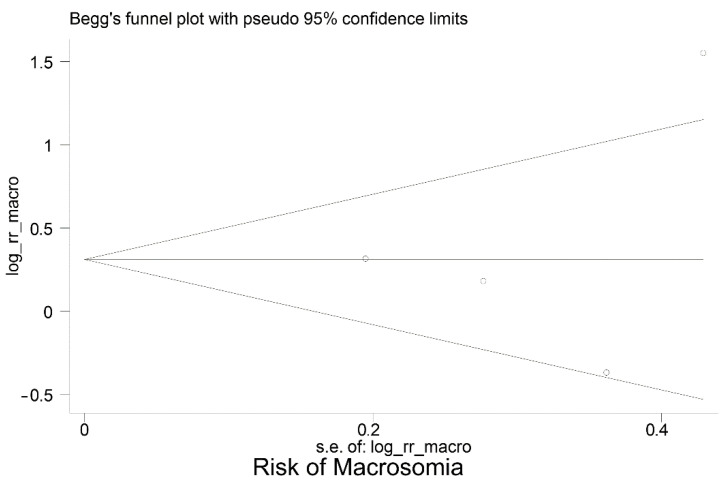
Funnel plot for publication bias—fetal macrosomia.

Table 3 summarises the data extracted from the eight studies included in the meta-analysis. Moreover, Figure 2 shows the meta-analyses of six studies reporting the relative risk of having an LGA baby in women with GDM who have high vs. normal or low HbA1c levels; the pooled relative risk (RR) was 1.70 (95% CI: 1.23 to 2.35), *p* = 0.001. This indicates the risk of LGA is significantly higher in women with high HbA1c levels, compared to women with normal or low HbA1c levels. However, the pooled RR for fetal macrosomia (Figure 3) was not significantly higher in women with high HbA1c compared to normal or low HbA1c, i.e., the pooled RR was 1.45 (95% CI: 0.80,2.63), *p* = 0.215. Between-study heterogeneity for LGA was (I-squared = 68.3%, *p* = 0.008) and for macrosomia (I-squared = 75.2%, *p* = 0.007). Egger’s tests (LGA *p* = 0.214 and fetal macrosomia *p* = 0.721) and funnel plots (Figure 4 and Figure 5) showed no indication of publication bias for the two meta-analyses, but due to the small number of studies there was limited power for this to be assessed. Due to the low number of studies, we could not perform any subgroup or meta-regression analyses to further explore the impact of between-study heterogeneity on estimated effect sizes.

### 3.3. Study Quality

Using the NIH quality assessment tool, most of the studies scored ten or above, which is considered as being of good quality [16]. A summary of the study quality assessment is provided in Supplementary Table S1.

## 4. Discussion

In this systematic review, we present novel data of a strong association between high HbA1c levels at 24–28 weeks of pregnancy and fetal macrosomia and LGA in 17,711 women with GDM. This study suggests a strong association between high HbA1c levels and large for gestational age or macrosomia in GDM pregnancies. Further, we observed that women with high HbA1c levels (5.5% or above) compared to women with low HbA1c levels (below 5%) were more likely to have fetal macrosomia or LGA babies.

Barquel et al. [17] stated that average third trimester HbA1c above 5.0% and excessive weight gain during pregnancy contributes to neonatal overweight and complications. Other authors, Xin et al., stated that a 1% increase in HbA1c level during pregnancy doubled the risk of having a large baby [22].

Mikkelsen et al. stated that HbA1c in late pregnancy is a good marker for identifying the risk of an LGA baby [23].

Two similar studies, Sweeting et al. [19] and Katon et al. [21], investigated a total of 2756 women with GDM. Together, they reported 424 LGA and 175 macrosomic babies when maternal HbA1c levels were 5% or above (total of 2015 women). In comparison, 97 LGA and 42 macrosomic babies were observed when maternal HbA1c levels were less than 5%. Barquel et al. [17] and Sweeting et al. [19] used the lowest cut-off HbA1c value of 5, whereas Xin et al. [22] used the highest cut-off value of 6.5. We also found that in all the included studies, the average maternal age was higher than 30.9 years and the average maternal pre-pregnancy BMI was greater than 24 kg/m^2^ (except in two studies). This observation implies that the mean maternal age and mean pre-pregnancy body weight could also contribute to fetal weight gain.

In recent years, smartphone apps have become popular and have been increasingly used to monitor glycaemic status in women with GDM, and studies have shown it to be of benefit in improving pregnancy outcomes [40,41,42]. Telemedicine, for example, and mobile phone apps, have also been used to monitor blood glucose levels closely. Moreover, once women were diagnosed with GDM, they would usually receive treatment, including dietary and lifestyle advice, as well as pharmacological interventions such as metformin and insulin. Despite all of these measures, we observed that the likelihood of fetal macrosomia or an LGA baby remained high in women with GDM.

The main strength of this systematic review and meta-analysis is the comprehensive search strategy; we studied a total of 17,711 pregnant women. Furthermore, the included studies were not confined to any particular geographic area or ethnic group. A limitation of our systematic review and meta-analysis was that there was no common cut-off value of HbA1c to demarcate high and low HbA1c levels. The cut-off HbA1c values ranged from 5 to 6.5. In a bid to address this issue, we attempted to obtain individual patient data, but in vain. Additionally, it was not possible to analyse the data according to ethnic variants, due to the limited number of studies and heterogeneity amongst the included studies.

## 5. Conclusions

Timely diagnosis and treatment of GDM are crucial in mitigating pregnancy complications, in particular fetal macrosomia and LGA babies, which lead to early induction of labour or caesarean delivery. This adds to the health and financial burden to the mother and the family and considerable costs to healthcare providers such as the National Health Services (NHS) in the UK. Therefore, it is essential to identify potential indicators for fetal macrosomia and LGA babies.

This is the most comprehensive and up to date meta-analysis investigating the association between HbA1c levels at 24–28 weeks of pregnancy and fetal macrosomia or LGA babies in women with GDM. The findings from this study suggest that high HbA1c levels (5.5% or above) between 24–28 weeks of pregnancy, is significantly associated with a future risk of fetal macrosomia or an LGA baby in women with GDM. Further research is needed to evaluate the utility of HbA1c levels in predicting the delivery of a baby with fetal macrosomia or LGA in pregnant women.

## Figures and Tables

**Table 1:** Summary of included studies (n = 24). The number in brackets is the standard deviation where reported. Braga et al. presented median value instead of mean.

Author	Year	Country	Total GDM	Macrosomia	LGA	Mean HBA1C	DX Criteria	Duration	Mean Age	BMI
**Alfadhil** [31]	2015	Saudi Arabia	292	10	-	5.77 (±0.82)	IADPSG	2011–2014	32.69 (±6.08)	32.30 (±6.66)
**Antoniou** [38]	2020	Switzerland	740	45	95	5.50 (±0.4)	IADPSG	2012–2017	32.80 (±5.5)	26.10 (±5.4)
**Barquel** [17]	2016	Spain	2037	-	126	5.20 (±0.4)	NDDG	1987–2008	33.00 (±4.0)	24.70 (±4.7)
**Braga** [35]	2019	Brazil	78	9	-	5.68	Carpenter & Coustan	2004–2005	31.00	27.80
**Buhary** [32]	2016	Saudi Arabia	177	31	-	6.58	WHO	2012–2013	31.88	31.31
**Capula** [30]	2013	Italy	148	3	10	5.28 (±0.29)	Two step procedure IWCGDM	2009–2010	33.4 (±4.8)	26.40 (±5.2)
**Dalfra** [28]	2011	Italy	1300	-	269	5.20	Carpenter & Coustan	2001–2007	33.48	24.90
**Gonzalez-quintero** [33]	2007	Miami, USA	3218	376	462	5.10–5.50	-	2001–2005	31.11	29.47
**Hu** [24]	2020	China	1155	108	112	5.36	ADP	2012–2017	31.30	22.62
**Kansu-celik** [36]	2019	Turkey	69	12		5.31 (±0.58)	Carpenter & Coustan	2010–2018	31.11 (±6.93)	27.43 (±5.49)
**Katon** [21]	2012	USA	502	210	210	5.70	-	2000–2010	31.00 (±5.4)	25.00
**Krstevska** [39]	2009	North Macedonia	180	37	-	6.23 (±1.2)	ADA	2006–2009	31.29	28.20 (±6.2)
**Liu** [25]	2020	China	81	3	-	4.98	WHO 13	-	31.95	22.02
**Mane** [34]	2017	Spain	22	3	4	5.90	ADA	2013–2015	33.81 (±4.87)	30.41 (±5.46)
**Mikkelsen** [23]	2011	Denmark	148	-	38	5.37	2-h 75 g OGTT	2007	32.57	28.86
**Olmos** [18]	2012	Chile	251	21	44	5.56	WHO	1998–2009	32.75	26.31
**Pintaudi** [29]	2018	Italy	2736	132	163	5.10 (±0.8)	Italian recommendation	2012–2015	36.60 (±5.10)	24.80
**Sweeting** [19]	2017	Australia	1805	161	411	5.30 (±0.5)	ADP	1991–2011	33.20 (±5.0)	24.00 (±5.1)
**Veres** [37]	2015	Romania	26	6	-	6.50	Carpenter & Coustan	2009–2011	31.31 (±4.47)	27.84 (±4.45)
**Wong** [20]	2017	Australia	1244	-	142	5.40 (±0.4)	ADIPS	2010–2014	31.60 (±5.2)	27.50 (6.9)
**Xin** [22]	2018	Singapore	202	-	21	5.99	WHO-2011	2012–2013	33.07 (±4.6)	-
**Xu** [26]	2019	China	1200	142	260	5.40 (±0.62)	IADPSG	2016–2017	30.90 (±4.2)	23.50 (±3.4)
**Zhao** [27]	2019	China	100	8	-	6.10–6.30	75 g OGTT	2014–2017	31.90	26.40

**Table 2:** Source of funding for the included studies (n = 23).

Study, Year	Source of Funding	Type of Study
**Alfadhil 2015** [31]	Supported by grant from King Abdulaziz City for Science and Technology, Riyadh, Saudi Arabia.	Prospective descriptive study
**Antoniou 2020** [38]	This study was sponsored by an unrestricted educational grant from NovoNordisk.	Prospective study
**Barquel 2016** [17]	None	Observational study
**Braga 2019** [35]	Supported by “Fundação do Amparo à Pesquisa do Estado do Rio de Janeiro” (Faperj)	Prospective, longitudinal and observational study
**Buhary 2016** [32]	None	Retrospective study
**Capula 2013** [30]	Not mentioned	Observational study
**Dalfra 2011** [28]	Not mentioned	Observational study
**Gonzalez-quintero 2007** [33]	Not mentioned	Retrospective cohort study
**Hu 2020** [24]	Supported by the National Natural Science Foundation of China under by Foundation for Innovative Research Groups of the National Natural Science Foundation of China; the National Key Research and Development Program of China; the Project of National Key Clinical Division of China, the Medical Scientific Research Foundation of Jiangsu Province of China; the Key Research and Development Program of Jiangsu Province of China; the Key Provincial Talents Program of Jiangsu Province of China; the Six talent peaks project of Jiangsu Province of China	Retrospective cohort study
**Kansu-Celik 2019** [36]	Not mentioned	Retrospective cohort
**Katon 2012** [21]	Funded by the University of Washington Department of Epidemiology.	Observational study
**Krstevska 2009** [39]	Nothing mentioned	Observational study
**Liu 2020** [25]	This study was funded by Sun Yat-Sen University Clinical Research 5010 Program and National Natural Science Foundation of China	Cohort study
**Mane 2017** [34]	Received no specific grant from any funding agency in the public, commercial, or not-for-profit sectors.	Prospective cohort study
**Mikkelsen 2011** [23]	Not mentioned	Cohort study
**Olmos 2012** [18]	Not mentioned	Observational study
**Pintaudi 2017** [29]	Not mentioned	Observational, retrospective, multicentre study
**Sweeting 2017** [19]	Not mentioned	Retrospective cohort
**Veres 2015** [37]	Not mentioned	Prospective cohort
**Wong 2017** [20]	None	Retrospective review
**Xin 2018** [22]	None	Retrospective study
**Xu 2019** [26]	Supported by grants from the National Natural Science Foundation of China Grant Award, National Key Research and Development Program of China, the Project of National Key Clinical Division of China, and the Medical Scientific Research Foundation of Jiangsu Province of China.	Retrospective Cchort study
**Zhao 2019** [27]	Not mentioned	Observational study

**Table 3:** Data extracted from the eight studies included in the meta-analysis.

*Author*	*Year*	*Total GDM*	*Number of LGA for Low HbA1c*	*Number of LGA for High HbA1c*	*Number of Low HbA1c*	*Number of High HbA1c*	*Cut-off HbA1c Value*	*Number of Macro Low HbA1c*	*Number of Macro High HbA1c*
Barquel [17]	2016	2037	20	95	526	1301	5	-	-
Xin [22]	2018	202	14	7	165	37	6.5	-	-
Sweeting [19]	2017	2254	89	410	449	1805	5	29	160
Wong [20]	2017	1244	55	87	675	569	5.4	-	-
Buhary [32]	2019	177	-	-	94	83	6.5	6	25
Olmos [18]	2012	251	-	-	202	49	6	30	14
Mikkelsen [23]	2011	148	18	20	97	51	5.6	-	3
Katon [21]	2012	502	8	14	292	210	5.7	13	15

## Data Availability

All data underlying this article are available in the article and in its online Supplementary Material. We will willingly share our knowledge, protocol and expertise when asked.

## Supplementary Materials

The following supporting information can be downloaded at: https://www.mdpi.com/article/10.3390/jcm12113852/s1, Supplementary Table S1—National Institute of Health (NIH) quality assessment tool for included studies (n = 23); Supplementary Table S2—Search strategy (Medline); Supplementary Table S3—PRISMA Checklist.
