# Supplementary material for: Association between HbA1c Levels and Fetal Macrosomia and Large for Gestational Age Babies in Women with Gestational Diabetes Mellitus: A Systematic Review and Meta-Analysis of 17,711 Women
Source: J Clin Med. 2023 Jun 5;12(11):3852. doi: 10.3390/jcm12113852 (PMC10253627; doi:10.3390/jcm12113852)
Supplement: Supplementary file 1 [file jcm-12-03852-s001.zip › Supplementary Tables 1 and 2.pdf]

Supplementary Table S1. National institute of health (NIH) quality assessment tool for included studies (n=23).

Author	*Q1*	*Q2*	*Q3*	*Q4*	*Q5*	*Q6*	*Q7*	*Q8*	*Q9*	*Q10*	*Q11*	*Q12*	*Q13*	*Q14*	*Total Score*	*Comment*
Alfadhil, 2015	Y	Y	Y	Y	Y	Y	Y	Y	Y	NR	Y	N/A	N/A	Y	11	Good
Antoniou, 2019	Y	Y	Y	Y	Y	Y	Y	Y	Y	Y	Y	N/A	N/A	Y	11	Good
Barquiel, 2016	Y	Y	Y	Y	Y	Y	Y	N/A	Y	NR	Y	N/A	NA	Y	10	Good
Braga, 2018	Y	Y	Y	Y	Y	Y	Y	Y	Y	NR	Y	N/A	N/A	Y	11	Good
Buhary, 2016	Y	Y	Y	Y	Y	Y	Y	Y	Y	NR	Y	N/A	N/A	Y	11	Good
Capula, 2013	Y	Y	Y	Y	Y	Y	Y	Y	Y	Y	Y	Y	N/A	Y	13	Good
Dalfra, 2010	Y	Y	Y	Y	Y	Y	Y	Y	Y	Y	Y	N/A	N/A	Y	13	Good
Gonzalez-Quintero, 2007	Y	Y	Y	Y	Y	Y	Y	Y	Y	NR	Y	N/A	N/A	Y	11	Good
Hu, 2019	Y	Y	Y	Y	Y	Y	Y	Y	Y	NR	Y	N/A	N/A	Y	11	Good
Kansu-Celik, 2019	Y	Y	Y	Y	Y	Y	Y	Y	Y	NR	Y	N/A	N/A	Y	11	Good
Katon,	Y	Y	Y	Y	Y	Y	Y	Y	Y	NR	Y	N/A	N/A	Y	11	Good
Krstevska, 2009	Y	Y	Y	Y	Y	Y	Y	Y	Y	NR	Y	N/A	N/A	Y	11	Good
Liu, 2020	Y	Y	Y	Y	Y	Y	Y	Y	Y	NR	Y	N/A	N/A	Y	11	Good
Mane, 2017	Y	Y	Y	Y	Y	Y	Y	Y	Y	NR	Y	N/A	N/A	Y	11	Good
Mikkelsen, 2011	Y	Y	Y	Y	Y	Y	Y	Y	Y	Y	Y	Y	N/A	Y	13	Good
Olmos, 2012	Y	Y	Y	Y	Y	Y	Y	Y	Y	Y	Y	NA	NA	Y	12	Good
Pintaudi, 2018	Y	Y	Y	Y	Y	Y	Y	Y	Y	Y	Y	N/A	N/A	Y	12	ood
Sweeting, 2017	Y	Y	Y	Y	Y	Y	Y	Y	Y	NR	Y	N/A	N/A	Y	11	Good
Veres, 2015	Y	Y	Y	Y	Y	Y	Y	Y	Y	NR	Y	N/A	N/A	Y	11	Good
Wong, 2017	Y	Y	Y	Y	Y	Y	Y	Y	Y	Y	Y	Y	N/A	Y	13	Good
Chong Xin, 2018	Y	Y	Y	Y	Y	Y	Y	Y	Y	NR	Y	N/A	N/A	Y	11	Good
Xu, 2019	Y	Y	Y	Y	Y	Y	Y	Y	Y	NR	Y	N/A	N/A	Y	11	Good
Zhao, 2019	Y	Y	Y	Y	Y	Y	Y	Y	Y	NR	Y	N/A	N/A	Y	11	Good

Supplementary Table S2. Search strategy (Medline).

Search | Journals | Books | Multimedia | My Workspace | What's New

▼ Search History (21) View Saved

# ▲	Searches	Results	Type	Actions	Annotations
1	Gestational diabetes mellitus.mp. or exp Diabetes, Gestational/	19344	Advanced	Display Results | More	
2	GDM.mp.	10234	Advanced	Display Results | More	
3	exp Pregnancy/ or Pregnancy diabetes mellitus.mp. or exp Pregnancy in Diabetics/	980328	Advanced	Display Results | More	
4	Hyperglycaemia in pregnancy.mp.	105	Advanced	Display Results | More	
5	pregnancy induced diabetes.mp.	18	Advanced	Display Results | More	
6	Fetal macrosomia.mp. or exp Fetal Macrosomia/	3590	Advanced	Display Results | More	
7	LGA.mp. or exp Birth Weight/	46771	Advanced	Display Results | More	
8	Large for gestational age.mp.	3098	Advanced	Display Results | More	
9	Overweight newborn.mp.	7	Advanced	Display Results | More	
10	macrosomia.mp.	5687	Advanced	Display Results | More	
11	macrosomia baby.mp.	0	Advanced	Save | More	
12	HbA1c.mp. or exp Glycated Hemoglobin A/	64932	Advanced	Display Results | More	
13	HaemoglobinA1c.mp.	13	Advanced	Display Results | More	
14	HemoglobinA1c.mp.	75	Advanced	Display Results | More	
15	Glycated hemoglobin A1c.mp.	1257	Advanced	Display Results | More	
16	Glycosylated hemoglobinA1c.mp.	8	Advanced	Display Results | More	
17	1 or 2 or 3 or 4 or 5	982931	Advanced	Display Results | More	
18	6 or 7 or 8 or 9 or 10 or 11	49461	Advanced	Display Results | More	
19	12 or 13 or 14 or 15 or 16	65316	Advanced	Display Results | More	
20	17 and 18 and 19	602	Advanced	Display Results | More	
21	limit 20 to (english language and humans)	522	Advanced	Display Results | More	

Contract
